# Effect of Nano-SiC Loading on Surface Discharge Performance of Polyimide at High-Frequency Electric Stress

**DOI:** 10.3390/polym17182526

**Published:** 2025-09-18

**Authors:** Ruoqing Hong, Qingmin Li, Huan Li, Qingming Xin

**Affiliations:** 1 State Key Laboratory of Alternate Electrical Power System with Renewable Energy Sources, North China Electric Power University, Beijing 102206, China; lqmeee@ncepu.edu.cn; 2 Electric Power Research Institute, China Southern Power Grid, Guangzhou 510663, China; lihuan3@csg.cn (H.L.); xinqm@csg.cn (Q.X.)

**Keywords:** nano-silicon carbide (SiC), polyimide (PI), high-frequency power transformers (HFPTs), surface and volume resistivity

## Abstract

This study targets insulation challenges in high-frequency power transformers (HFPTs), which are an integral part of the high-voltage, high-capacity isolated DC/DC converter under development for offshore renewable energy systems. We propose a nano-silicon carbide (SiC)-doped polyimide (PI) winding insulation strategy to enhance discharge resistance and thermal stability under high-frequency electric stress. Experimental results show that 10 wt% SiC doping significantly improves insulation performance, extending failure time from 17 to 50 min and reducing maximum discharge amplitude by 76%, owing to enhanced charge trapping and interfacial polarization suppression. Surface and volume resistivity measurements further confirmed the improvement; at 120 °C, the 10 wt% SiC composite maintained high surface resistivity 3.30 × 10^14^ Ω and volume resistivity 1.41 × 10^15^ Ω·cm, significantly outperforming pure PI. In contrast, 20 wt% SiC, though still resistive, showed reduced stability due to agglomeration and interfacial defects, with a surface resistivity of 2.07 × 10^14^ Ω and degraded dielectric performance. Dielectric analysis revealed that 10 wt% SiC suppressed dielectric constant and loss across the frequency range, while 20 wt% SiC exhibited increased values at high frequency. These results highlight 10 wt% SiC as an optimal formulation for HFPT winding insulation.

## 1. Introduction

High-frequency power transformers (HFPTs) are integral components of high-voltage, high-capacity isolated DC/DC converters. They play a vital role in modern power systems by enabling precise voltage and current regulation, thereby ensuring high power density and optimal power quality [1,2]. Polyimide (PI) has emerged as the preferred insulation material for HFPTs due to its outstanding dielectric properties, excellent thermal resistance, and superior chemical stability [2,3,4,5]. However, the unique operational conditions of HFPTs pose significant challenges to conventional PI films. The high-frequency sinusoidal waveforms typical of HFPTs intensify both skin and proximity effects in the magnetic core, leading to increased insulation losses and notable temperature rise during operation [6,7].

These thermal effects are further aggravated by electrical stress. Notably, the surface flashover strength of insulating materials is considerably lower than their bulk breakdown strength over equivalent distances, making HFPTs particularly susceptible to creeping discharge [8,9]. This susceptibility is especially pronounced around insulation defects, such as sharp edges or material imperfections, where local electric field enhancement can trigger discharge activity. The combination of thermal degradation and electrical discharges accelerates insulation aging and compromises transformer reliability over time [10,11].

Among these electrical stress phenomena, surface discharge represents a major failure mechanism. Since the surface flashover field strength is significantly lower than the volume breakdown strength for similar gap distances, surface discharge often becomes the primary cause of insulation degradation and early failure [12,13]. Studies have shown that the inception voltage of surface discharges is strongly influenced by the microstructure of the dielectric material as well as the electrode geometry [14]. Under high-frequency square wave excitation, surface discharge exhibits complex dynamics—initially showing a decrease and then an increase in both discharge amplitude and repetition rate [15]. Additionally, investigations under nanosecond pulse voltages have revealed considerable differences between single-pulse and repeated-pulse discharge behaviors [16,17]. The progression of surface discharge typically occurs in distinct stages that are closely related to the material properties of the insulating film [18,19].

In recent years, efforts to enhance the performance of PI films under high-frequency conditions have largely focused on nano-doping strategies. These modifications have shown success in improving discharge endurance [20,21]. Alternative methods based on molecular structure modifications—such as incorporating bulky side chains or introducing asymmetry into the polymer backbone—have also demonstrated improvements in the optical and dielectric behavior of PI materials [22,23]. For instance, PI films containing 40% phenyl thioether (PSI40) have shown excellent discharge endurance (153 min), attributed to optimized dielectric loss characteristics and modified microstructure. However, further investigation is needed to fully understand how varying thioether concentrations impact the long-term performance of these materials [24].

Similarly, nano-SiO_2_ doping has been found to significantly improve the discharge resistance of PI films, with 10% doping (Si10) extending the material’s lifetime by a factor of 3.4 compared to undoped PI (Si0). These performance gains are linked to enhancements in resistivity, optical absorption, and trap distribution, which collectively mitigate electrical-thermal aging and discharge-induced damage [25]. Recently, silicon carbide (SiC) has gained increasing attention in the development of polyimide (PI)-based nanocomposites, although the material is still in the optimization phase. Incorporating SiC nanoparticles into PI films has been shown to significantly enhance high-frequency insulation performance by reducing dielectric loss, suppressing partial discharge activity, minimizing surface damage, and extending the aging lifetime [26].

Silicon carbide (SiC) exists in several polytypes—most notably cubic 3C (β-SiC) and hexagonal 4H/6H (α-SiC)—whose bandgaps and defect landscapes differ and underpin their performance in harsh, high-frequency environments. Recent studies highlight β-SiC synthesis routes on Si substrates and associated microstructural features, while reviews of SiC devices and sensors emphasize the material’s wide bandgap, high defect tolerance, and stability at elevated temperature and radiation fluxes. Additionally, polytype-dependent chemical durability has been documented, with strong resistance to both acidic and alkaline media. These attributes support the use of nano-SiC as a multifunctional filler to aid thermal management, field homogenization, and discharge suppression in polymer dielectrics [27,28,29].

While nanofiller-doped polyimides such as SiO_2_, and TiO_2_ have been widely reported to enhance dielectric properties, their effectiveness is often limited under high-frequency operating conditions due to low thermal conductivity and insufficient suppression of localized discharge activity. In contrast, SiC offers a unique combination of high thermal conductivity, wide bandgap semiconducting behavior, and mechanical robustness, which enables it to simultaneously dissipate localized Joule heating, reduce charge injection, and improve structural stability during electro-thermal cycling. These attributes address a critical gap in the literature: whereas conventional oxide fillers improve permittivity and breakdown strength, they do not adequately resolve the coupled challenges of thermal management and partial discharge mitigation encountered in high-frequency power transformer (HFPT) environments. Therefore, the present work highlights the novel role of SiC as a multifunctional filler that not only improves dielectric performance but also enhances discharge endurance under accelerated high-frequency stress [30,31].

To further address these challenges, this study investigates the high-frequency surface discharge behavior of PI films modified with nano-sized silicon carbide (SiC) particles. A dedicated experimental platform was developed to assess discharge characteristics at the air–PI interface, including amplitude, frequency, phase, and morphological features. Microstructural analysis combined with SEM elucidate how SiC incorporation affects the development of surface discharges. The findings offer valuable theoretical insights for material modification and insulation structure design in HFPTs, aiming to enhance discharge endurance while preserving low dielectric loss in high-frequency applications.

## 2. Material Synthesis Experiment Scheme

### 2.1. Materials and Film Preparation

The composite polyimide (PI) films were synthesized using the following chemical components: pyromellitic dianhydride (PMDA, C_10_H_2_O_6_, ≥99% purity), 4,4′-diaminodiphenyl ether (ODA, C_12_H_12_N_2_O, ≥98% purity), N,N-dimethylacetamide (DMAC, C_4_H_9_NO, ≥99% purity), and silicon carbide (SiC, 99.9% purity). All reagents were obtained from Macklin china and used without further purification.

To investigate the effects of nano-SiC on insulation performance, SiC was introduced at two loadings 10%, and 20% by molar ratio to PMDA based on optimization of breakdown strength and surface discharge resistance Table 1. In a typical synthesis, 0.015 mol of ODA and the designated amount of nano-SiC (average particle size ~40 nm) were dispersed in 25 mL of purified DMAC in a 50 mL beaker. The mixture was ultrasonicated at 80 °C for 1 h to ensure homogeneous dispersion of the nanoparticles.

Following ultrasonic treatment, the dispersion was transferred to a three-neck flask and cooled to 0–5 °C using an ice–water bath. Under continuous mechanical stirring, 0.0153 mol of PMDA was gradually added. As the dianhydride was introduced, the solution began to thicken, indicating the formation of polyamic acid (PAA). Once all PMDA had been added, the mixture was allowed to react at 45 °C for 3 h to complete the polycondensation, yielding a viscous, gray PAA solution.

**Figure 1:**
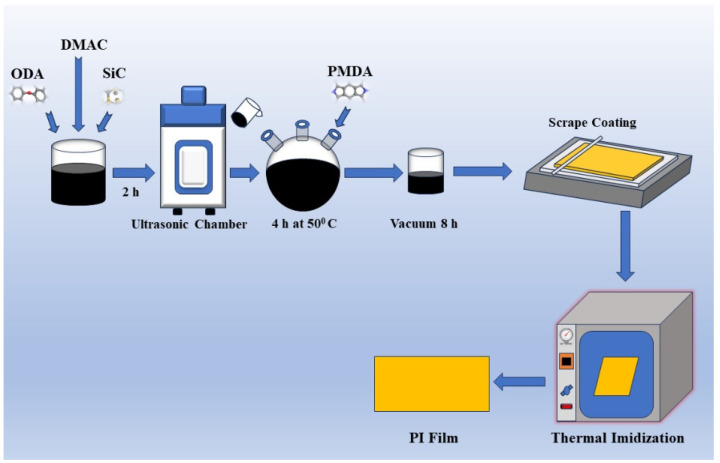
Preparation steps of the composite PI films.

Before film casting, the PAA solution was degassed under vacuum to remove trapped air. It was then cast onto thoroughly cleaned glass substrates, pretreated with ethanol and deionized water, and spread evenly using a glass scraper to form a uniform wet film layer. The entire process for preparing the composite PI films is illustrated in Figure 1.

The coated polyamic acid (PAA) films underwent thermal imidization using a stepwise heating program in a high-temperature oven. The temperature was gradually increased following the sequence: 80 °C for 1 h, 100 °C for 1 h, 150 °C for 1 h, 200 °C for 1 h, 250 °C for 30 min, and finally 300 °C for 30 min. Upon completion of the imidization process, the glass substrates were removed from the oven and allowed to cool naturally to room temperature. The resulting polyimide (PI) films were then released by immersing the glass plates in deionized water, producing flexible, free-standing films with an average thickness of approximately 50 μm. To prepare the films for subsequent characterization, they were dried in an oven to remove residual moisture and cleaned with anhydrous ethanol to ensure surface purity.

### 2.2. Dielectric Constant and Loss Trends in PI/SiC Nanocomposites

**Figure 2:**
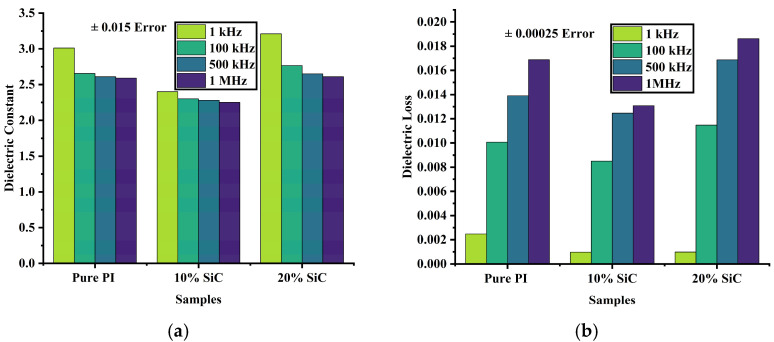
Dielectric properties: (**a**) Dielectric constant. (**b**) Dielectric loss.

The dielectric constant and dielectric loss behaviors of pure polyimide (PI) and its composites doped with 10% and 20% silicon carbide (SiC) nanoparticles were systematically evaluated across four frequencies using GB/T 31838.6-2021: 1 kHz, 100 kHz, 500 kHz, and 1 MHz. For pure PI, the dielectric constant exhibited a gradual decline with increasing frequency, decreasing from 3.011 at 1 kHz to 2.59 at 1 MHz. This frequency-dependent reduction is typical of polymeric dielectrics, where dipolar polarization mechanisms become less effective at higher frequencies, leading to lower relative permittivity. In comparison, the 10% SiC-doped PI composite consistently demonstrated lower dielectric constant values across all frequencies, ranging from 2.4 at 1 kHz to 2.25 at 1 MHz as shown in Figure 2a. This suppression of permittivity indicates that SiC nanoparticles enhance insulation behavior by disrupting dipole alignment and reducing interfacial polarization, likely through improved trap distribution and microstructural modification, thereby minimizing dielectric loss under high-frequency electrical stress.

Conversely, the 20% SiC-doped PI composite displayed a distinct trend. At 1 kHz, the dielectric constant reached 3.21 higher than that of pure PI, suggesting the presence of pronounced interfacial polarization due to the increased concentration of SiC nanoparticles. However, as the frequency increased, the dielectric constant dropped more sharply, reaching 2.61 at 1 MHz and approaching the value observed for pure PI.

Pure PI exhibited increasing loss with frequency as shown in Figure 2b, rising from 0.00247 at low frequency to 0.01688 at 1 MHz, reflecting greater energy dissipation due to lagging dipolar relaxation. The 10% SiC-doped sample, however, showed a significantly lower dielectric loss of 0.00097 at low frequency and remained below that of pure PI across the full frequency range, with a maximum value of 0.01308 at 1 MHz. This reduction indicates improved insulation efficiency, likely due to the SiC-induced suppression of charge mobility and interruption of conduction pathways. In contrast, the 20% SiC-doped PI film exhibited a similarly low dielectric loss at low frequency (0.00098), but the loss increased more rapidly with frequency, reaching 0.01861 at 1 MHz, higher than both pure PI and the 10% composite. This suggests that excessive SiC content may lead to agglomeration or interfacial defects, promoting localized polarization and increased energy dissipation.

### 2.3. Temperature-Dependent Resistivity of PI/SiC

**Figure 3:**
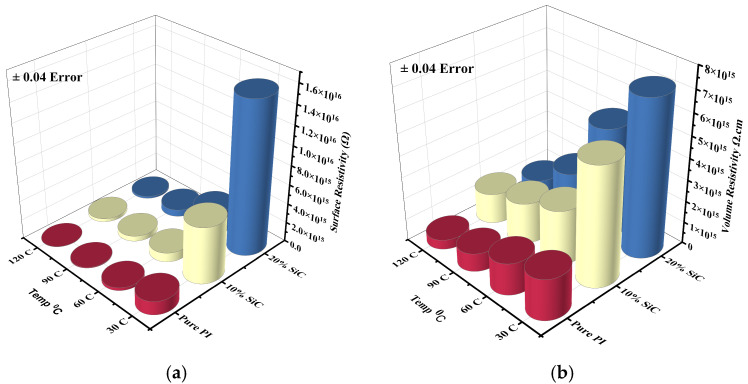
PI/SiC resistivities: (**a**) Surface Resistivity. (**b**) Volume Resistivity.

The surface resistivity of pure polyimide (PI) and its composites containing 10% and 20% silicon carbide (SiC) was measured at 30 °C, 60 °C, 90 °C, and 120 °C using a ZC36 high insulation resistance tester, as shown in Figure 3a, to evaluate their thermal stability and electrical insulation performance under elevated temperatures. At 30 °C, all samples exhibited high surface resistivity, with pure PI showing 1.41 × 10^15^ Ω, while the 10% and 20% SiC-doped samples exhibited significantly higher values of 5.89 × 10^15^ Ω and 1.57 × 10^16^ Ω, respectively. This indicates that the incorporation of SiC nanoparticles enhances surface insulation properties at room temperature, with the 20% loading providing the highest resistivity due to likely improvements in barrier effects and microstructural uniformity. As the temperature increased to 60 °C, surface resistivity declined in all samples, reflecting thermally activated charge carrier mobility. Pure PI dropped to 3.06 × 10^14^ Ω, while the 10% and 20% SiC samples maintained higher resistivity values of 9.42 × 10^14^Ω and 2.43 × 10^15^ Ω, respectively. At 90 °C, the decline continued, with pure PI falling to 7.46 × 10^13^ Ω, while the SiC-doped composites retained higher levels: 5.50 × 10^14^ Ω for 10% SiC and 7.07 × 10^14^ Ω for 20% SiC. At 120 °C, surface resistivity further decreased, with pure PI reaching 1.76 × 10^13^ Ω, while the 10% and 20% SiC composites remained comparatively stable at 3.30 × 10^14^ Ω and 2.07 × 10^14^ Ω, respectively.

The volume resistivity of pure polyimide (PI) and its composites containing 10% and 20% silicon carbide (SiC) was measured at 30 °C, 60 °C, 90 °C, and 120 °C to evaluate their bulk insulation stability under thermal stress, as shown in Figure 3b. At 30 °C, pure PI exhibited a volume resistivity of 1.91 × 10^15^ Ω·cm, while the 10% and 20% SiC-doped composites showed significantly enhanced values of 5.52 × 10^15^ Ω·cm and 7.22 × 10^15^Ω·cm, respectively. This improvement at room temperature indicates that SiC incorporation effectively increases the bulk resistance of the polymer matrix by introducing interfacial barriers that impede charge transport. As the temperature increased to 60 °C, a general decline in volume resistivity was observed due to thermally activated charge carriers. Pure PI dropped to 1.49 × 10^15^Ω·cm, whereas the 10% and 20% SiC composites retained higher resistivities of 2.55 × 10^15^ Ω·cm and 5.10 × 10^15^ Ω·cm, respectively. At 90 °C, the downward trend continued: pure PI decreased to 8.49 × 10^14^ Ω·cm, while the 10% and 20% SiC composites remained more stable, at 1.91 × 10^15^ Ω·cm and 2.12 × 10^15^ Ω·cm, respectively. Even at 120 °C, the SiC-filled composites maintained a clear advantage: pure PI dropped further to 4.49 × 10^14^ Ω·cm, whereas the 10% and 20% SiC samples exhibited higher resistivities of 1.41 × 10^15^ Ω·cm and 8.82 × 10^14^ Ω·cm, respectively.

These results clearly demonstrate that SiC doping significantly enhances the thermal stability of volume resistivity in PI, with 10% SiC providing the most balanced performance across the temperature range. Such improvements underline the potential of PI/SiC nanocomposites as robust insulating materials for high-temperature and high-frequency electrical applications.

### 2.4. Surface and Cross-Sectional SEM of PI/SiC Composites

**Figure 4:**
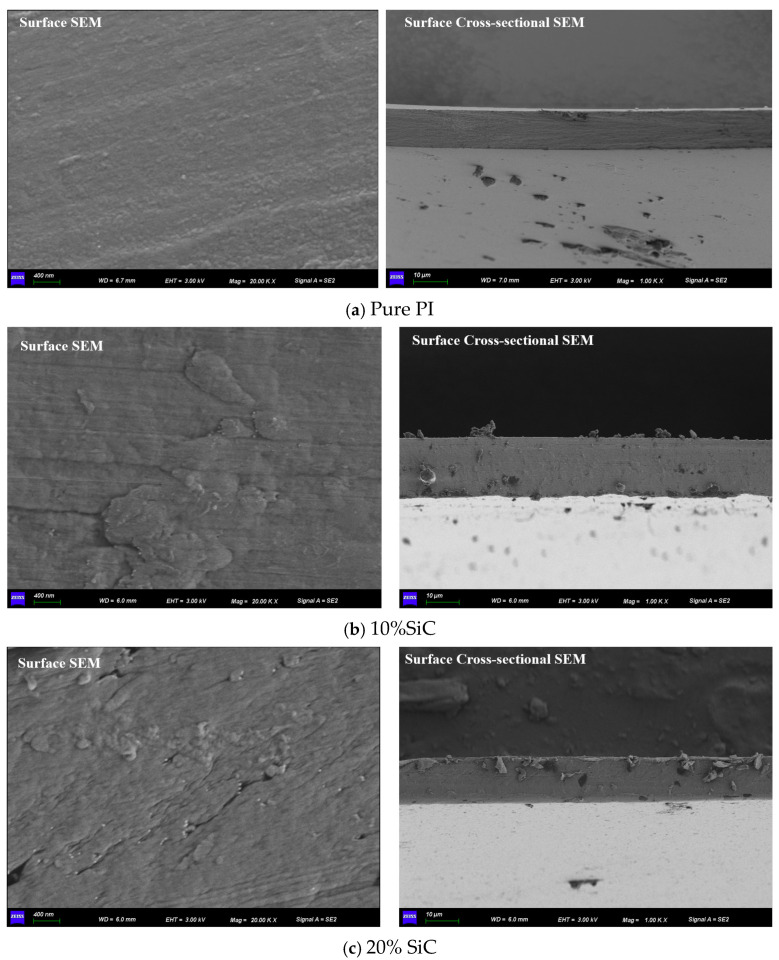
Surface and Cross-Sectional SEM of PI/SiC Composites.

The surface SEM image of pure PI, captured using a ZEISS Sigma 360 (Frankfurt, Germany), reveals a relatively smooth and homogeneous morphology, with no visible particulates or significant surface irregularities. The cross-sectional image further confirms the dense and uniform structure with good film continuity and no apparent voids or defects. This indicates a well-formed, compact matrix typical of neat polyimide films, which contributes to its good insulating properties shown in Figure 4a.

The surface of the 10% SiC-doped PI shows the presence of well-dispersed particles with mild surface roughness shown in Figure 4b. The roughness increases compared to pure PI, indicating successful incorporation of SiC nanoparticles. No large agglomerates are visible, which suggests that the dispersion at this concentration remains relatively uniform. In the cross-section, small particulates can be observed embedded in the matrix, and the interface appears well bonded, with no major gaps or delamination. This microstructure is favorable for improved trap density and thermal conduction pathways while maintaining structural integrity.

The 20% SiC sample exhibits a much rougher and more irregular surface morphology, shown in Figure 4c. The SEM surface image shows noticeable clusters and uneven dispersion of SiC particles, suggesting nanoparticle agglomeration at higher loading. The cross-sectional image reveals more pronounced particulate accumulation and a slightly less uniform matrix compared to the 10% sample. This may lead to localized stress points and defects, which could negatively affect the dielectric performance and mechanical reliability. The interfacial bonding also appears slightly compromised due to the higher filler content.

**Figure 5:**
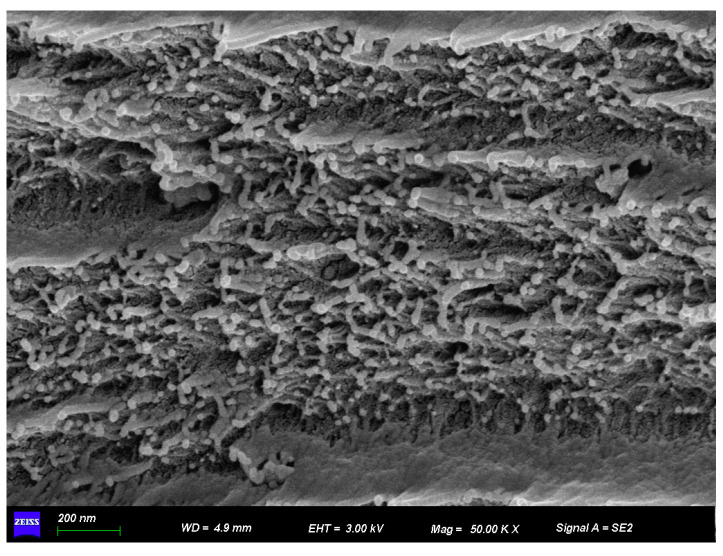
Surface of (PI) composite doped with silicon carbide (SiC) nanoparticles.

The high-magnification SEM image reveals the detailed surface morphology of a polyimide (PI) composite doped with silicon carbide (SiC) nanoparticles shown in Figure 5. The structure appears dense yet highly porous, with numerous granular features embedded within the matrix. These bright, irregularly shaped particles are indicative of SiC, dispersed throughout the polymer. While the dispersion is generally effective, some degree of clustering is also visible, suggesting partial nanoparticle agglomeration.

## 3. Surface Discharge Experimental Setup

**Figure 6:**
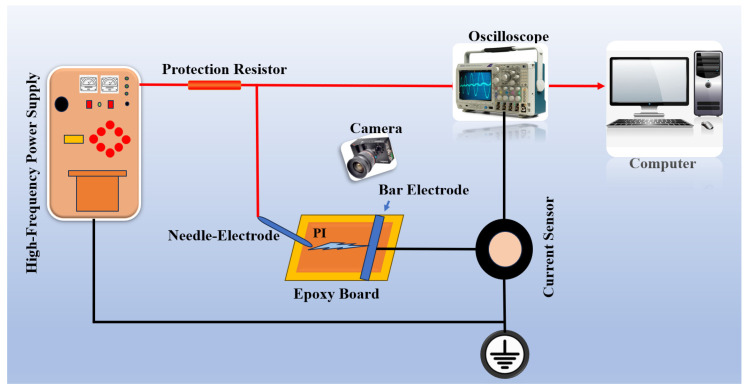
Surface discharge experimental setup.

The experimental setup for high-frequency surface discharge is shown in Figure 6. A high-frequency power supply generates a continuous sinusoidal voltage ranging from 5 to 50 kHz with a peak value of 0–20 kV. A needle–plate electrode configuration is used, where the needle electrode is 25 mm long, 1 mm in diameter, with a 4 mm tip radius, placed 10 mm from the plate at a 45° angle to the sample surface. The test material is a 5 cm × 5 cm, 50 μm-thick polyimide (PI) film fixed on an epoxy board.

Surface discharge is measured using the pulse current method, with the ETS-93686(ETS-Lindgren, Cedar Park, TX, USA) high-frequency current sensor (bandwidth: 300 kHz–100 MHz). Signals are captured by a Tektronix MDO3024 oscilloscope (Tektronix, Inc., Beaverton, OR, USA). A high-speed camera records the discharge process above the electrode. Data acquisition and storage are handled in real time through a USB interface and LabVIEW.

### 10% SiC Optimizes PI’s Discharge Resistance: PRPD Study

**Figure 7:**
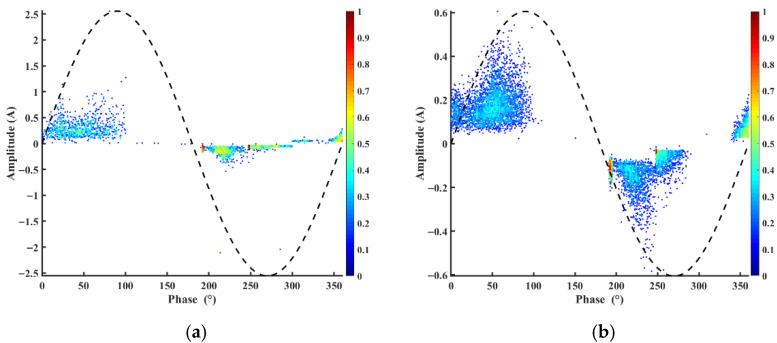
Phase-Resolved Partial discharge patterns (**a**) Pure PI (**b**) 10%SiC.

The PRPD results reveal significant differences in the partial discharge behavior and insulation lifetime of pure polyimide (PI) and its SiC nanocomposites, as shown in Figure 7. Pure PI exhibits a short lifetime of 17 min with a high maximum discharge amplitude of 2.53, indicating poor resistance to partial discharge degradation due to uncontrolled charge accumulation and weak trapping mechanisms. In contrast, the 10% SiC-doped PI demonstrates a dramatically improved lifetime of 50 min—nearly three times longer than pure PI—along with a much lower discharge amplitude of 0.6, highlighting the role of SiC nanoparticles in suppressing discharge intensity by enhancing charge dissipation and interfacial barriers.

The observed 50 min lifetime refers to the insulation failure time recorded under accelerated high-frequency electro-thermal stress testing. Although this time scale is much shorter than the expected service life in practical applications (which typically spans years), it serves as a useful comparative benchmark for evaluating material improvements under consistent and controlled conditions. Importantly, the modified polyimide/SiC composites not only extended the failure time but also produced a 76% reduction in maximum partial discharge amplitude compared with pure polyimide. This suppression of discharge activity can be attributed to several mechanisms: (i) charge scattering and trapping at nanoparticle interfaces, which suppress local electric field intensification; (ii) improved thermal conductivity and field homogenization provided by SiC, which reduce localized heating and prevent premature breakdown; and (iii) structural densification, where nanoparticles fill free-volume regions and hinder defect growth. Together, these effects demonstrate that the incorporation of SiC nanoparticles both delays the onset of failure and reduces the severity of degradation, offering valuable insight into degradation mechanisms and guiding the design of more durable insulation systems for high-voltage, high-frequency applications.

However, the 20% SiC composite shows severe degradation, with a drastically reduced lifetime of just 5 min, suggesting that excessive filler loading leads to agglomeration-induced defects, accelerating insulation failure. Unfortunately, insufficient PRPD data is available for the 20% SiC sample to fully analyze its discharge patterns, but the shortened lifetime strongly implies compromised dielectric performance due to structural inhomogeneity. These findings underscore the critical balance between SiC content and dispersion quality in optimizing polyimide’s insulation properties.

**Figure 8:**
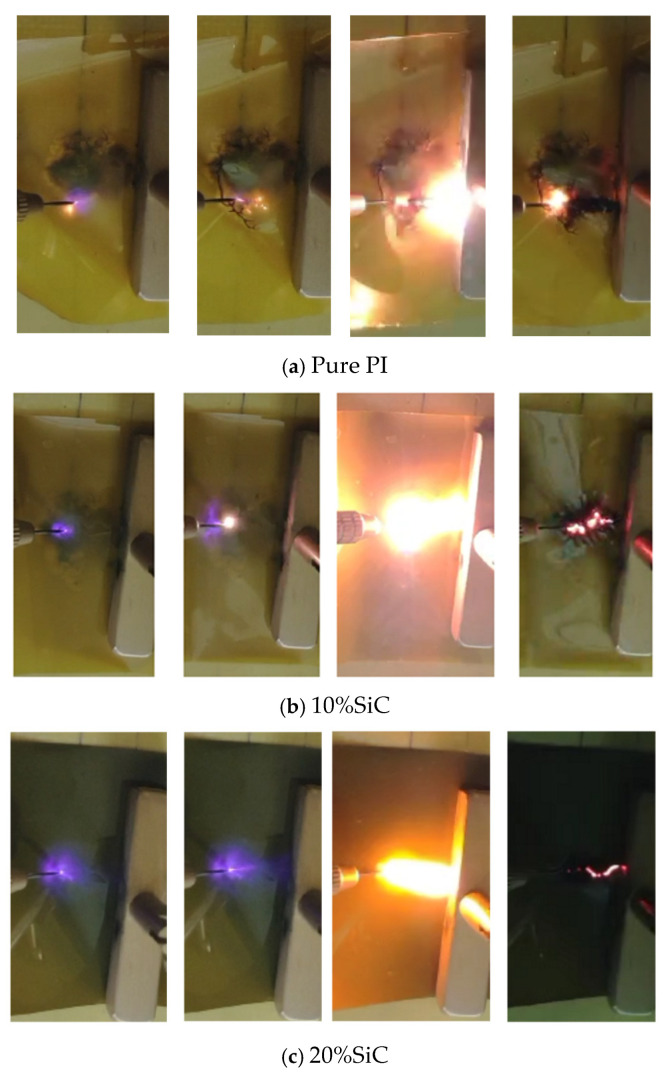
Development of Surface flashover.

Figure 8 demonstrates how silicon carbide (SiC) doping influences surface flashover development in polyimide (PI). Pure PI exhibits a steady but moderate flashover progression, while 10% SiC-PI shows improved resistance due to charge-scattering effects. At 20% SiC, flashover suppression is more pronounced but may become erratic if nanoparticle agglomeration creates weak spots. The shapes of the curves likely reflect these mechanisms: a smooth trend for pure PI, a steeper/interrupted rise for 10% SiC, and a multiphase response for 20% SiC. This aligns with the known trade-off between filler concentration and dielectric homogeneity in nanocomposites.

## 4. Conclusions

The integration of nano-silicon carbide (SiC) into polyimide (PI) films presents a promising strategy for addressing insulation challenges in high-frequency power transformers (HFPTs), which are core components of high-voltage, high-capacity isolated DC/DC converters. At an optimal loading of 10 wt%, SiC nanoparticles are uniformly dispersed within the PI matrix, resulting in significantly enhanced surface and volume resistivity, reduced dielectric constant and loss, and a substantial improvement in insulation failure time—from 17 to 50 min. These improvements are attributed to improved charge trapping, interfacial polarization suppression, and microstructural uniformity.

In contrast, excessive SiC loading (20 wt%) leads to nanoparticle agglomeration and interfacial defects, which deteriorate dielectric properties and accelerate insulation breakdown despite relatively high resistivity levels. SEM analysis confirms this trend, showing well-dispersed SiC at 10 wt% and clustering at 20 wt%. The 10 wt% SiC composite achieves the best balance between dielectric stability, discharge endurance, and thermal reliability, making it a strong candidate for HFPT winding insulation under high-frequency electric stress.

Future work should explore multi-phase nanofiller systems and advanced dispersion techniques to further optimize insulation performance while mitigating the adverse effects of excessive filler content.

## Figures and Tables

**Table 1:** Preparation Information.

No.	PMDA (mol)	ODA (mol)	SiC (mol)	DMAC
1	0.0153	0.015	0%	25 mL
2	0.0153	0.015	10%	25 mL
3	0.0153	0.015	20%	25 mL

## Data Availability

Data is contained within the article.
